# A Rare Case of Antiphospholipid Syndrome With Concomitant Antithrombin III Deficiency: A Case Report

**DOI:** 10.7759/cureus.51555

**Published:** 2024-01-02

**Authors:** Husam Barham, Munther W Alshyoukhi, Hani Siaj, Raed Masalma, Wesam Tamimi, Ali H Khalilia, Omar Almur, Mohammad Jaber

**Affiliations:** 1 Internal Medicine, Faculty of Medicine and Health Sciences, An-Najah National University, Nablus, PSE; 2 Internal Medicine, Palestine Medical Complex, Ramallah, PSE; 3 Internal Medicine, Hadassah Medical Center, Jerusalem, ISR; 4 Radiology, Faculty of Medicine and Health Sciences, An-Najah National University, Nablus, PSE; 5 Surgery, Palestine Medical Complex, Ramallah, PSE

**Keywords:** heparin, warfarin, thrombophilia, anticoagulation, antithrombin iii deficiency, antiphospholipid syndrome

## Abstract

Antithrombin (AT) deficiency and antiphospholipid syndrome (APS) are distinct but potentially overlapping disorders with significant implications for thrombosis. We present a case of a 28-year-old male with hereditary AT deficiency who subsequently developed primary APS. Despite the challenges of overlapping symptoms and anticoagulation therapy, a careful diagnostic approach revealed the coexistence of these rare conditions. The patient was successfully managed with long-term anticoagulation, hydroxychloroquine, and other supportive measures. This case underscores the importance of comprehensive laboratory testing, especially when managing patients with pre-existing anticoagulation needs.

## Introduction

Antithrombin (AT) is one of the most important factors in regulating the coagulation cascade. It works by inhibiting thrombin primarily alongside other activated proteases of the coagulation system. AT deficiency is a rare autosomal dominant disease that represents an increased risk for thrombosis, especially in the venous system, as arterial system thrombosis is less reported [1].

Antiphospholipid syndrome (APS), also known as Hughes syndrome, is an autoimmune disorder that is characterized by various thromboembolic events that can lead to multi-organ failure and early fetal loss in pregnancy [2]. Although APS was heavily linked with a diagnosis of systemic lupus erythematosus (SLE), it is now dealt with as a different entity; the misconception stems from the fact that early cases of APS were reported as cases of hemorrhage in previously diagnosed SLE patients [3].

Here, we report a 28-year-old male with a medical history of hereditary AT deficiency, who developed primary APS and was successfully treated with long-term anticoagulation and hydroxychloroquine therapy.

## Case presentation

A 28-year-old male patient presented to the emergency room on the 6th of July 2022 complaining of coughing large amounts of bright red blood associated with shortness of breath, which started suddenly at 10 am on the day of presentation. Past medical history was significant for hereditary AT deficiency, which was diagnosed 12 years ago when he presented to the hospital complaining of shortness of breath and was diagnosed to have pulmonary embolism and right leg deep vein thrombosis. The patient was treated with intravenous (IV) heparin and then switched and discharged on warfarin 10 mg once daily with monthly international normalized ratio (INR) testing keeping it at the range of 2.0-3.0, which is the therapeutic range for patients on anticoagulant therapy (normal is 1.0).

The patient reported no trauma, fever, night sweats, weight loss, anorexia, change or loss of appetite, fatigue, chest pain, palpitations, nausea, dysphagia, melena, hematochezia, seizure, blurred vision, muscle weakness, bone pain, or joint pain. The patient had no history of smoking, alcohol consumption, or non-steroidal anti-inflammatory drug use.

On physical examination, the patient was conscious, alert, and oriented to time, place, and person, with a Glasgow Coma Scale score of 15/15. He was afebrile with a pulse of 124 beats per minute, blood pressure of 138/82 mmHg, oxygen (O2) saturation of 92% on a 10-liter nasal cannula, and a respiratory rate of 30 respirations per minute. Lung auscultation revealed bilateral fine crackles up to mid-lung zones and his heart sounds were normal with no murmurs or added sounds. The abdomen was soft with no tenderness, guarding, rigidity, and normal bowel sounds.

Table 1 describes the initial laboratory values for the patient. INR was 3.8 on presentation and the patient received 5 mg of vitamin K and was admitted immediately to the intensive care unit (ICU), where he received five units of fresh frozen plasma and IV fluids and was put on a high-flow nasal cannula for O2 support. An echocardiography was done and showed no abnormalities.

**Table 1:** Initial laboratory values upon admission to the intensive care unit.

Blood test	Result	Reference range	Unit
Hemoglobin	17.0	13.5-17	g/dL
Hematocrit	49.1	43.5-53.7	%
Red blood cells	5.85	4.69-6.13	M/uL
Mean cell hemoglobin concentration	34.6	31-35	g/dL
Mean cell hemoglobin	29.1	27-31.2	pg
Mean cell volume	83.9	80-100	fL
Platelets count	338	150-450	K/uL
White blood cells	9.2	4.6-11	K/uL
Neutrophils	5.8	2.5-7.0	K/uL
Lymphocytes	3.1	0.7-4.8	K/uL
Monocytes	0.3	0.2-0.8	K/uL
Aspartate aminotransferase	132	0-50	u/l
Blood urea nitrogen	11.39	6-20	mg/dl
Creatinine	0.88	0.7-1.2	mg/dl
Alanine transaminase	168	0-41	u/l
Random blood sugar	115	74-110	mg/dl
Potassium	4.0	3.5-5.3	mEq/L
Sodium	143	135-145	mEq/L
Chloride	110	98-110	mEq/L
Calcium	8.91	8.6-10	mEq/L
Activated partial thromboplastin time	103.9	25-35	s
Prothrombin time	41.2	11-15	s
Internationalized normalized ratio	3.8	1.0	-
Thyroid-stimulating hormone	0.77	0.35-4.94	mIU/L
Bilirubin	2.306	0-1.2	mg/dl
Creatine kinase	485	0-190	u/l
Lactate dehydrogenase	359	207-414	u/l
C-reactive protein	23.4	0-5	mg/L
Troponin I	0.004	0-0.029	ng/ml
Fibrinogen	369	200-400	mg/dl
Complement III	113	90-180	-
Complement IV	20.4	10-40	-

**Figure 1:**
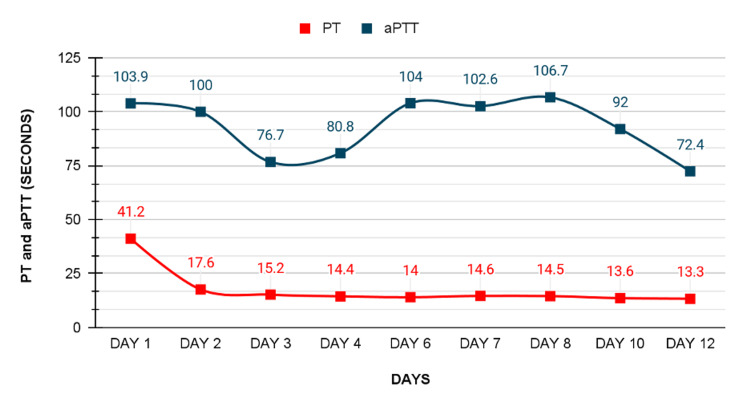
Timeline of PT and aPTT levels in seconds over 12 days. Day one represents admission day with a PT value of 41.2 seconds and aPTT of 103.9 seconds, both parameters consistently decrease, reaching a nadir and normal values on day 12 with a PT value of 13.3 seconds and an aPTT value of 72.4 seconds. PT: prothrombin time; aPTT: activated partial thromboplastin time.

**Figure 2:**
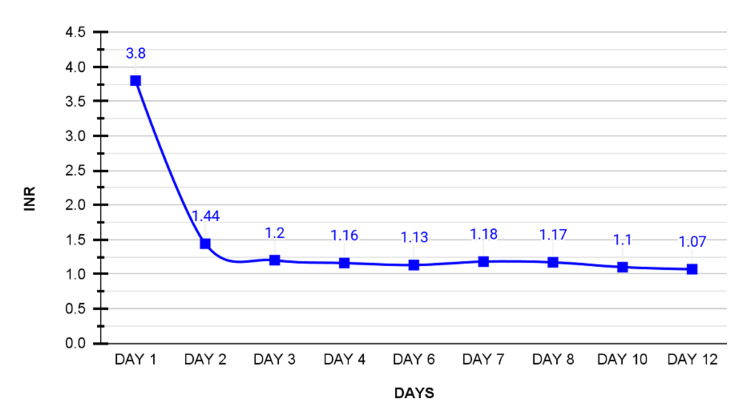
Timeline of INR levels over 12 days Day one represents admission day with the initial INR value of 3.8, subsequent days manifest a consistent decrease observed with a value of 1.07 on day 12. INR: international normalized ratio.

On the second day of admission, the patient felt better, and the bleeding stopped completely, INR dropped to 1.44, and hemoglobin dropped to 12.2 g/dL (normal range: 13.5-17.0 g/dL). The patient was still on O2 support. Activated partial thromboplastin time (aPTT) was 100 seconds, so a mixing study was ordered. Figures 1, 2 describe the changes in INR, prothrombin time (PT), and aPTT throughout the 12 days of his stay in the ICU.

On the third day, the patient showed no improvement in his clinical condition, and a pulmonary computed tomography pulmonary angiography (CTPA) was ordered to rule out pulmonary embolism, which showed no abnormalities. The mixing study showed no correction of aPTT; therefore, immunological profile laboratory tests were ordered, and the results came back positive for anticardiolipin antibody, lupus anticoagulant, anti-beta-2-glycoprotein-I antibody, antinuclear antibody (ANA), and anti-double stranded DNA (anti-dsDNA) antibody. A provisional diagnosis of SLE alongside APS was made based on the Sydney classification criteria. The patient was started on enoxaparin 80 mg subcutaneously twice daily and IV methylprednisolone 1 mg once daily for five days.

On the sixth day, the patient showed good improvement, and O2 support was stopped. Hydroxychloroquine 200 mg twice daily was added to his treatment regimen. On the eighth day, he finished his methylprednisolone regimen and was switched to oral prednisolone 40 mg once daily. On the 12th day, the patient was in good condition, so a decision was made to discharge him on the following drugs: oral prednisolone 40 mg once daily, subcutaneous enoxaparin 100 mg twice daily, oral pantoprazole 40 mg twice daily, and oral hydroxychloroquine 200 mg twice daily. A follow-up clinic appointment was scheduled in a week.

Upon the first follow-up appointment, the patient was well and had no symptoms, his INR was 1.2 and his aPTT was 71 seconds. He was kept on the same drugs except for prednisolone, which was tapered down gradually by 5 mg per week until reaching a complete cessation and subsequently stopped pantoprazole when the complete cessation of prednisolone was reached, and a follow-up appointment in two weeks was scheduled. After two weeks, the patient was still well and in good condition, INR was 2.3, and aPTT was 70 seconds, and follow-up appointments were scheduled monthly. After three months of the initial presentation and admission to the ICU, the patient returned for a follow-up appointment, and an immunological study was ordered to confirm the diagnosis of primary APS. Lupus anticoagulant, anticardiolipin antibody, and anti-beta-2-glycoprotein-I antibody came back positive, which confirmed the diagnosis of APS based on the Sydney classification criteria for the diagnosis of APS. The patient did not meet the Systemic Lupus International Collaborating Clinics (SLICC) criteria, which is used in our center for a definite diagnosis of SLE. Therefore, the diagnosis of primary APS was made.

## Discussion

This case presents a unique clinical scenario of a 28-year-old patient diagnosed with hereditary AT deficiency at the age of 13, who subsequently developed primary APS. Hereditary AT deficiency is a relatively uncommon autosomal dominant genetic disorder with a prevalence of 1:2000 to 1:3000. It has two types: type 1 quantitative deficiency and type 2 qualitative deficiency [4].

APS is an autoimmune multi-systemic disease that increases the risk of pregnancy morbidity and vascular thrombosis, especially at the sites of the lower limbs and cerebral arterial circulation [5]. It is diagnosed according to the revised Sydney classification criteria for the APS (Table 2). It is based on the presence of at least one clinical manifestation (vascular thrombosis and/or pregnancy morbidity) in the setting of persistently positive of at least one antiphospholipid antibody. Laboratory testing must be positive on two separate occasions at least 12 weeks apart to confirm persistence [3]. The APS antibodies are autoantibodies that are directed against phospholipid-binding proteins [5]. The coexistence of these two uncommon conditions in this patient highlights the importance of careful observation and adds to the complexity of the diagnosis.

**Table 2:** The revised classification criteria for APS diagnosis (Sydney criteria). Adapted from [3]. Antiphospholipid syndrome is diagnosed when at least one clinical manifestation is present alongside persistently positive one of the Laboratory criteria. APS: antiphospholipid syndrome; IgG: immunoglobulin G; IgM: immunoglobulin M; ELISA: enzyme-linked immunosorbent assay; GPL: IgG phospholipid units; MPL: IgM phospholipid units.

Clinical criteria	Laboratory criteria
(1) Vascular thrombosis: One or more clinical episodesof arterial, venous, or small vessel thrombosis, in any tissue or organ. Thrombosis must be confirmed by objective validated criteria (i.e., unequivocal findings of appropriate imaging studies or histopathology). For histopathologic confirmation, thrombosis should be present without significant evidence of inflammation in the vessel wall.	(1) Lupus anticoagulant (LA) present in plasma, on two or more occasions at least 12 weeks apart, detected according to the guidelines of the International Society on Thrombosis and Hemostasis.
(2) Pregnancy morbidity: One or more unexplained deaths of a morphologically normal fetus at or beyond the 10th week of gestation, with normal fetal morphology documented by ultrasound or by direct examination of the fetus, or one or more premature births of a morphologically normal neonate before the 34th week of gestation because of (i) eclampsia or severe pre‐eclampsia defined according to standard definitions or (ii) recognized features of placental insufficiency, or three or more unexplained consecutive spontaneous abortions before the 10th week of gestation, with maternal anatomic or hormonal abnormalities and paternal and maternal chromosomal causes excluded.	(2) Anticardiolipin (aCL) antibody of IgG and/or IgM isotype in serum or plasma, present in medium or high titer (i.e., >40 GPL or MPL, or >the 99th percentile), on two or more occasions, at least 12 weeks apart, measured by a standardized ELISA.
	(3) Anti‐β_2_ glycoprotein‐I antibody of IgG and/or IgM isotype in serum or plasma (in titer >the 99th percentile), present on two or more occasions, at least 12 weeks apart, measured by a standardized ELISA.

The management of APS and AT deficiency focuses on preventing episodes of thrombosis with long-term anticoagulation while minimizing the risk of hemorrhage from prophylactic anticoagulants. Vitamin K antagonists and low molecular weight heparin (LMWH) are the backbone for preventing such episodes, especially in patients who have previously developed thrombosis [4,6]. This shared treatment modality, while effective, adds a layer of difficulty to the whole clinical picture.

When a physician is looking after a patient with one thromboembolic disease, they could easily overlook signs of another due to the existing need for anticoagulation, as in this patient who already was taking warfarin, which masked all symptoms of APS. This overlap focuses on the dangers of relying only on visible symptoms. Thoroughly conducting laboratory tests is crucial to distinguish between thromboembolic diseases, as the application of the Sydney Classification Criteria was pivotal in diagnosing primary APS in this case.

In patients diagnosed with APS who fail to respond to warfarin therapy, recommendations are extended to add low-dose aspirin, a statin, as it inhibits tissue factor expression induced by antiphospholipid antibody, hydroxychloroquine, as it lowers venous thromboembolic events in patients with primary APS, low-weight molecular heparin, or a combination of these regimens [7].

When we consider this instance, it becomes clear that medicine is constantly evolving. Navigating through the unique mix of rare conditions that this patient faces reminds us that there is always more to learn.

## Conclusions

This case emphasizes the diagnosis of primary APS in a 28-year-old male with a past medical history of hereditary AT deficiency. Successful treatment involved anticoagulation and hydroxychloroquine. This scenario highlights the evolving nature of medical understanding and the vital role of thorough diagnostics in unraveling complex clinical presentations.
